# Using Semiautomated WhatsApp Messages for Daily Stress Measurements: Integrated Usability and Feasibility Study

**DOI:** 10.2196/84032

**Published:** 2026-03-11

**Authors:** Janika Thielecke, Maartje Bakhuys Roozeboom, Irene Niks, Elsbeth de Korte, Sadegh Shahmohammadi

**Affiliations:** 1 Work and Heath, The Netherlands Organization for Applied Scientific Research (TNO), Sylviusweg 71, Leiden, 7415 NC, The Netherlands; 2 Department Clinical-, Neuro-, and Developmental Psychology, Faculty of Behavioral and Movement Sciences, Vrije Universiteit Amsterdam, Amsterdam, The Netherlands

**Keywords:** work stress, whatsApp, chatbot, digital stress assessment, monitoring, artificial intelligence, AI, journalling, diary study, feasibility, usability

## Abstract

**Background:**

Stress is a key determinant of health outcomes and may influence work performance. Questionnaire-based assessments of stress are typically broad and retrospective. Daily stress measurements via smartphones offer more granular, real-time data but have adherence issues. Using an already established communication medium (WhatsApp) and a more conversational style assessment might improve adherence and help collect more detailed insights into (work) stress, underlying stressors, and countering energy sources.

**Objective:**

This study focuses on the usability and feasibility of semiautomated voice- and text-messages (with and without emojis) via WhatsApp as a method to collect daily data on experienced work stress, stressors, and energy sources.

**Methods:**

A sample of 210 workers was recruited via social media and participated in a 10-workday diary study using semiautomated WhatsApp messages to rate daily stress, stressors, and energy sources. Questions (with and without emojis) were presented by a chatbot as text messages with clickable buttons (multiple-choice questions; MC) or with instructions to answer with either a voice or a text message. The study used an experimental design with 4 groups: (1) week 1 voice, week 2 text/MC with emojis; (2) week 1 voice, week 2 text/MC without emojis; (3) week 1 text/MC, week 2 voice with emojis; (4) week 1 text/MC, week 2 voice without emojis. Pre- and poststudy web-based questionnaires assessed demographics, familiarity with voice messages, and usability, including participants’ preference for research studies. Open answers were coded using artificial intelligence, and the number of stressors or energy sources was compared across the 3 collection methods (MC, voice, and text messages) to determine if the amount and quality of information collected differ per method within participants.

**Results:**

A total of 158 workers completed at least 80% of scheduled conversations. The sample was predominantly women(170/210, 81%), highly educated (173/210, 82%), and a slight majority worked part-time (109/210, 52%). Mean adherence to the daily schedule was very high (mean of 95%). The postquestionnaire revealed a strong preference for MC and text over voice messages, mostly due to ease and convenience in a variety of situations. The number of stressors per week was approximately 3 times higher in the MC-condition than in the voice condition, even though average stress levels per week did not differ significantly within participants. The number of energy sources was comparable between open answers in the voice and text conditions, but voice messages consisted of more words.

**Conclusions:**

Collecting (work) stress data via semiautomatic WhatsApp messages is a feasible method with low effort for participants. Usability ratings indicated a strong preference among participants for MC and text messages over voice messages. Future research should explore usability in more diverse samples and in direct comparison to traditional assessment methods.

## Introduction

Stress is a fundamental aspect of human life and plays an important role across various domains, including health and occupational settings. Stress is not only a key determinant of mental and physical health outcomes [1-4] but also influences work performance [5], productivity [6,7], cognitive processes [8,9], and overall well-being of employees [10].

Especially in the workplace, understanding stress and contributing factors such as long-term stressors, daily hassles, and sources of energy is critical for developing and applying effective strategies that support the employee’s well-being. To be able to act on work-related stress in a timely manner, an important precondition is that work-related stress and contributing factors can be measured close to real-time and with enough detail to inform actions [11,12]. In the next sections, we first briefly discuss the advantages and disadvantages of different stress measurement methods in this regard. We then suggest a novel way to assess (work-related) stress and influencing factors with a conversational style assessment. We then continue by explaining how this study tests the usability and feasibility of this new approach.

Measuring stress in general, including in the workplace, is a challenge, as (work-related) stress is a highly subjective experience, depending on an individual’s perception and appraisal of (potential) stressors [13]. While physiological, presumably more objective stress measures in the workplace are on the rise [14,15], the most common way to assess (work-related) stress today remains through standardized self-report questionnaires consisting of multiple items [13,16].

Commonly used questionnaires to assess (work-related) stress rely on one-time, retrospective questions covering a period of several days or weeks [13]. However, these approaches are less suitable for uncovering daily stress and hassles [13]. That is, retrospective recall is considered prone to biases, especially over longer periods of time, as individuals may misremember or reinterpret their experiences based on their current emotional state [17]. At the same time, one-time questionnaires are likely to capture a summary evaluation of stressful moments or could be impacted by a single, extreme event but fail to capture the context in which stress was experienced, making it hard to act on.

Alternatives to retrospective one-time questionnaires are diary studies and ecological momentary assessments (EMAs), which are both used to capture individuals’ experiences in or near real-time with short questionnaires often consisting of single items [18]. This is tackling the problem of recall bias. With the spread of technology, these methods have become more widely applicable with specialized software and applications to facilitate web-based data collection. Traditional diary studies typically ask participants to record their thoughts, feelings, or behaviors at the end of the day or at scheduled intervals, either as open questions or short questionnaires. Similarly, EMA is a more structured and intensive version of diary studies, often involving multiple prompts for short questionnaires throughout the day to capture momentary experiences. Repeated or daily measures in these studies explicitly aim to capture the different situations in which stress might be experienced. This gives much more granular and actionable insights compared with a once-a-week or monthly measure.

Nevertheless, both traditional questionnaires and EMA face some shared challenges. Accessing context factors is taxing, as closed multiple-choice (MC) questions, while quick and convenient, might not capture a situation completely. Open questions, on the other hand, are time-intensive for both participants and analysts, especially if asked multiple times per day. Additionally, both types require time and motivation from participants to answer the questions. While traditional questionnaires require time and motivation only once or occasionally, adherence to daily questionnaires likely requires greater motivation from participants. Adherence to EMA is one of its biggest challenges, as research has shown that many participants quickly uninstall (research-related) apps, reducing long-term engagement and adherence [19,20]. On the other hand, EMA can provide more direct insights and moments of reflection for the participants [21].

To address the above-mentioned challenges (ie, collecting real-time contextual information and engaging participants in daily measurements) while keeping the advantage of real-time EMA, we suggest focusing on a more conversational style stress measurement using familiar and established conversation platforms like WhatsApp.

WhatsApp is currently the most popular messaging application globally, with 3 billion active users [22] and in the Netherlands, with 13.3 million active users [23]. Given its integration into daily life, WhatsApp makes it easier to take part in research studies and answer questions. It therefore has an advantage compared with the use of special research applications. In a conversation-like assessment via WhatsApp, questions are asked by a chatbot and can be either written chats, voice chats, clickable buttons, or a mixture of those. Providing more detailed answers or situational information to a “conversation partner” (ie, a chatbot) could feel more natural and engaging for participants, as suggested by the trend to discuss mental health topics with large language models (LLMs) like ChatGPT [24]. Being able to record voice messages instead of having to type the answer might also reduce the burden of providing open answers and increase overall adherence.

Concerning the integration of open answers from participants, the emergence of LLMs also makes it easier to process potentially large amounts of open-ended responses, whether in text or spoken form. Advanced natural language processing (NLP) techniques allow researchers to efficiently analyze and categorize responses, identifying themes [25,26] and sentiment [27,28] associated with stress.

Semiautomated chatbots using emojis or GIFs for engagement are already used in marketing for customer satisfaction surveys and interactive support [29-31], showing the potential of using this medium for measurement and intervention. In addition, the low-threshold communication might reach individuals who are less frequently included in research studies, such as young people or people with a lower education [21].

The use of communication apps for work-related stress measurements also aligns with broader trends in digital health and workplace well-being initiatives. Many organizations are exploring digital tools to monitor employee stress and well-being, with the goal of developing targeted interventions and support systems [12,32]. If harnessing commonly used communication channels for work-related stress assessments proves to be feasible, this approach could be further developed into a personalized and low-effort monitoring and support system for employees.

This study adds valuable knowledge on the usability and feasibility of communication apps like WhatsApp as a tool for daily data collection in the pursuit of low-threshold (work-related) stress monitoring and interventions. In terms of usability, we are interested in who would be willing to participate in a study on work-related stress using this new data collection method (sample description). Another open question concerns how individuals perceive this method and their preferences regarding question types (ie, text, voice message, or MC buttons). In terms of feasibility, we need a better understanding of how this method performs in terms of uptake and adherence and the quality of information collected. In this study, we therefore investigate adherence rates as well as the effect of emojis on adherence, as emojis are a typical and easy-to-implement feature of mainstream WhatsApp communication. To evaluate the quality of the data collected, we will look at (1) potential differences in the number of reported contextual factors (ie, stressors and energy sources) per data collection method, (2) the number of words used to describe energy sources using voice or text messages, and (3) the relationship between the number of reported stressors and participants’ stress ratings.

We formulated the following research questions for our exploratory study on usability (RQ1) and feasibility (RQ2 and RQ3), focusing on employees who took part in a 10-day diary study:

- RQ1: What is the user experience of participants over a 2-week period?
- RQ2: What is the level of adherence (percentage of daily measurements completed) in relation to the different collection methods and the use of emojis in WhatsApp?
- RQ3: What is the quality of the data in the different collection methods regarding the number of stressors and energy sources reported, the number of words used, and the relation of stressors to the stress rating?

## Methods

### Study Design

**Figure 1.**
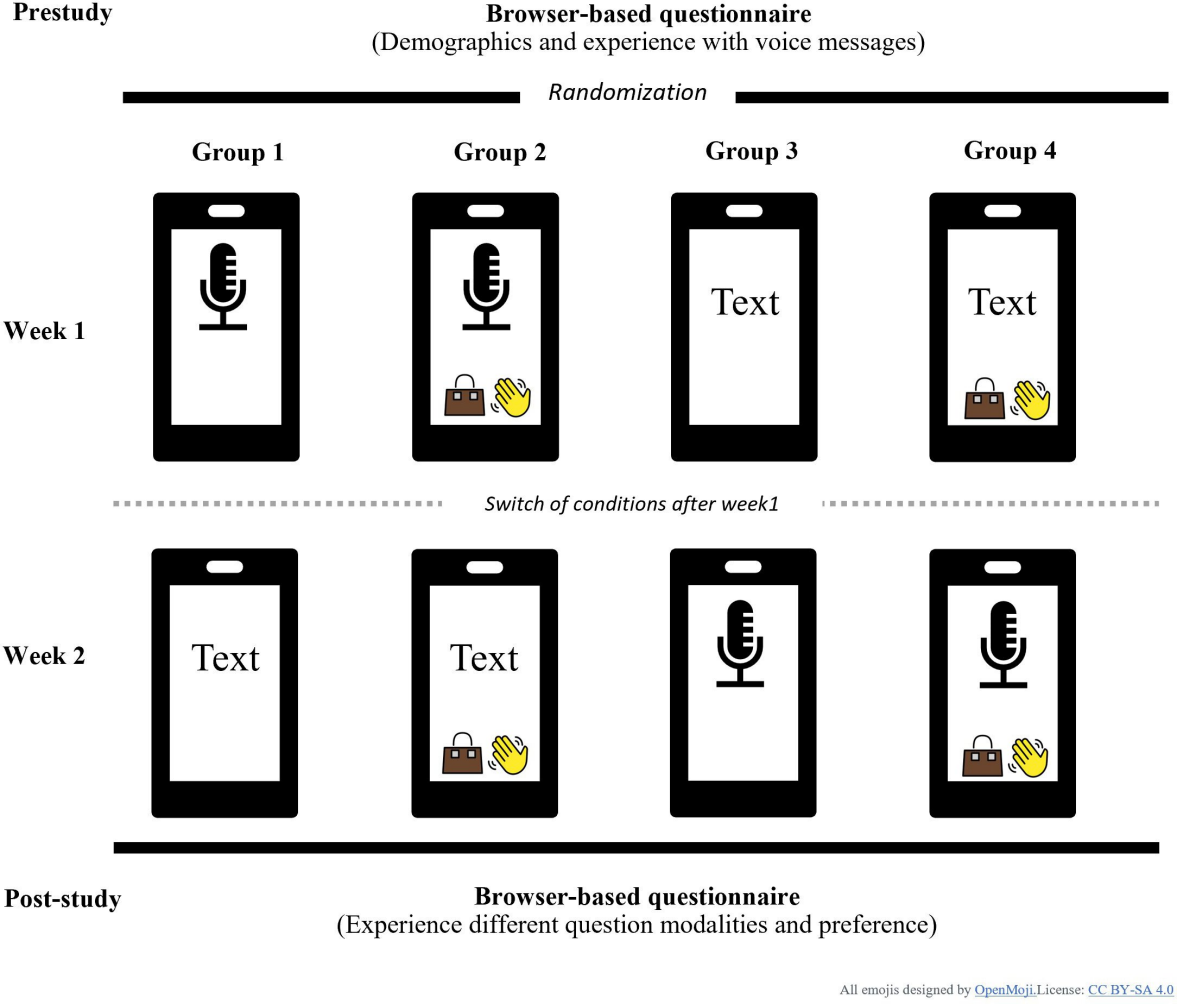
Study design (emojis adapted from [33], which is published under Creative Commons Attribution 4.0 International License [34]).

To answer the research questions, we adopted an experimental study design in a 2-week diary study on work-related stress and its contributing factors, using a semiautomated WhatsApp chatbot that asked 4 questions at the end of each workday. Each participant experienced both answer formats: voice messages or a combination of text messages and MC questions. Answer methods were switched after the first week: Half of the participants began answering questions using voice messages (voice condition) and would use MC messages (operationalized as template buttons) and text messages in the second week (text/MC-condition). The other half of the participants followed the reverse order. Additionally, both conditions were presented either with or without emojis, resulting in 4 experimental groups (Figure 1). For emojis, we chose more technical emojis, such as the battery symbol for the question on energy givers and the microphone for voice messages, to avoid adding emotional stimuli. Demographic data and user experience were collected via browser-based questionnaires before and after the daily WhatsApp interactions. Participants joined the study in 6 weekly waves between September and October 2024.

All daily messages were sent semiautomatically using the marketing tool [35], connected via the research organization’s Meta account to a WhatsApp Business account created solely for this study. Eazy is an omnichannel marketing platform that is advertised to streamline and automate communication and support marketing campaigns; it is not routinely used in research contexts. Eazy programmed the 4 different experimental conditions as simple automatic, rule-based chatbots to interact with the participants. When participants answered a message, this triggered the next messages in the conversation script. The chatbot always instructed participants on how to respond, either by providing buttons with predefined answers or by requesting a text or voice message (Multimedia Appendix 1). Participants were informed at the beginning of the study that no human would monitor or engage in the WhatsApp conversation. All messages exchanged between the chatbot and participants were end-to-end encrypted.

### Ethical Considerations

The research proposal was approved by The Netherlands Organization for Applied Scientific Research (TNO) Internal Review Board (registration number: 2024‐071) after reviewing the research design, privacy considerations, ethical aspects, and the potential burden and risks to participants. All participants provided informed consent via a web-based survey (Survalyzer) prior to enrolling in the WhatsApp conversation. Data from the browser-based questionnaires were linked to WhatsApp conversations using phone numbers, which were subsequently removed and replaced with pseudonyms. All analyses were conducted on pseudonymized data. Participants did not receive direct compensation but could enter a lottery for 50 Amazon gift vouchers (1 × €100, 3 × €50, 46 × €20; conversion rate on payment date: 1€ = US $1.0580) if they completed at least 80% of daily measurements.

### Procedure

We recruited unsystematically on social media for 6 weeks. Recruitment started on LinkedIn because of its closeness to the work setting. There, we published and shared posts from the research team members and promoted one of the posts through a paid ThoughtLeader campaign through TNO as the responsible research organization. After a limited response, we collaborated with TNO’s Marketing and Communication department, which professionally organized paid advertising campaigns on Meta platforms (Instagram and Facebook) using the official TNO account. Social media campaigns were not specifically targeted at specific groups, as we were interested in the broad working population. All social media posts included a picture of a phone, a short description of the study (duration, topic, inclusion criteria, lottery), highlighted the use of voice data, and included a direct link to the Survalyzer sign-up platform. People clicking on the link were shown detailed participant information, including contact information to reach the study team (via email). Participants who wanted to participate could digitally sign an informed consent. Participants were then asked to fill in a short prequestionnaire on demographics, use of voice messages, and the telephone number they wanted to use. At the start, participants were informed how to disable automatic messaging by sending a stop word in the WhatsApp chat. On the final page, participants were automatically randomized and either shown a QR code to initiate the WhatsApp conversation (waves 1‐2) or informed that the study team would add them manually using their telephone number (waves 3‐6). During the study, the procedure was changed from QR code initiation to manual addition due to technical difficulties in renewing QR codes promptly.

The semiautomated messages were scheduled separately for each wave by the study team, starting on Thursdays. Messages were delivered Mondays through Fridays at 6 PM local time to the participants’ own devices. After the first week, participants received a message informing them that they were halfway through the study and that the response method would now change. At the end of the study, participants were sent a link to an evaluation questionnaire and received a reminder one week later.

### Randomization and Blinding

Randomization was done fully automated in Survalyzer. Participants were randomly assigned to one of 4 groups. Data collectors were not blinded because telephone numbers from waves 3 to 4 had to be manually transferred from Survalyzer to the marketing tool by the study team. Participants were blinded to their assigned condition.

### Participants

Participants had to be 18 years or older, living in the Netherlands, and native Dutch speakers without a speech impairment. They were further required to be employed for at least 20 hours or 3 days per week, not to work night shifts, already have an existing WhatsApp account, and provide informed consent. Participants confirmed these criteria when providing informed consent.

### Measurements

All data was collected through the web-based questionnaire tool Survalyzer (pre- and postquestionnaire), and through WhatsApp messages managed via Eazy.

### Prequestionnaire

In the prequestionnaire, we assessed participants’ gender (, woman, man, other), age (≤24, 25‐34, 35‐44, 45‐54, 55+ years), number of weekly workdays (3 d, 4 d, 5 d), highest educational attainment (Dutch equivalents of low, medium, and high education), and frequency of voice message use on WhatsApp (never, occasionally, sometimes, often).

### Daily Measurements

Daily measurements always started with the question of whether the participant had worked that day. If not, no further questions followed, and participants were sent a closing message wishing them a pleasant day. On a workday, the same 3 questions about experienced stress, stressors, and energy sources were asked, but the response methods differed across experimental conditions.

Experienced stress was measured consistently across all days using a single item adapted from the SMS-based stress assessment by Arapovic-Johansson et al [36]. Participants were asked: “Stress is a state in which you feel tense, restless, nervous, or anxious. Have you experienced stress today?.” Participants responded using a drop-down menu ranging from 1 (“not at all”) to 10 (“very much”).

The questions on experienced work-related stressors and energy sources were self-developed as there were no validated single-item measurements available. The question on work-related stressors was answered either in an MC-format or with a voice message. For the MC-condition, we chose ten broad and commonly reported work-related stressors [37,38] to accommodate a broad target group (the amount of work; time pressure; emotional burden; constantly having to process information; negative atmosphere; work impediments/problems with systems or processes; uncertainty about tasks/task distribution or planning; conflicts or threats; lack of support or appreciation from supervisors; overlapping of work and private life; other [open answer]). Each stressor was presented separately with “yes” or “no” response options. In the voice condition, the question was phrased as follows, without examples: “What stressors did you experience today in your work? Share it with us in a voice message!.” The question on energy sources was always an open question to be answered either by text or voice message (“Where did you draw energy from today or in which area did you have a good experience? Please describe this briefly in a [voice or text] message.”).

### Postquestionnaire

For the postquestionnaire, we measured participants’ preferences and experiences with different communication methods using self-created items. We asked which method they found more pleasant (“Which method did you find more pleasant to use?”), easier (“Which method did you find easier to use?”), and safer (“Which method did you find safer to use?”) to use, and which allowed them to express themselves best (“With which method could you best express your experiences or opinions?”). For each question, participants could choose between “voice messages,” “text messages,” “multiple-choice questions,” and “no preference.” We further asked if they experienced any technical problems (“Did you experience any technical problems?”) with any of the methods. Three open-ended questions asked participants what they liked or disliked about each method (“What did you like or dislike about [text messages / voice messages /multiple-choice questions]? When or in what situation would you like to use [text messages / voice messages /multiple-choice questions]?”).

Concluding, we asked what kind of methods participants would generally prefer in research (“If you were asked to participate in a study, which method would you prefer?”) with the following options: “WhatsApp questionnaires (multiple-choice questions),” “WhatsApp text messages,” “WhatsApp voice messages,” “A (phone) conversation,” “Online questionnaires in a browser (like this one),” “A questionnaire in a special research app,” “Group discussion,” “Other, namely: …").

Participants who wished to enter the lottery as a token of appreciation for their effort were asked to enter their name and email address.

### Statistical Analysis 

To answer research questions 1 on participants’ experiences, we present descriptive statistics from the pre- and postquestionnaire as well as stress ratings from the daily measurements. For research question 2, we compared adherence rates between the different conditions. Adherence was defined as the proportion of working days on which participants responded in the WhatsApp conversation, relative to the number of working days they reported in the prequestionnaire.

To test whether the use of emojis was related to the adherence, we conducted independent Wilcoxon rank tests between the emoji groups (groups 1 and 3) and the nonemoji groups (groups 2 and 4) for overall adherence and adherence per week.

To answer research question 3 concerning the differences in the number of named work-related stressors and energy sources in total, we compared groups using paired Wilcoxon signed-rank tests. The same method was used to compare the average number of words used in the text and voice answers on energy sources.

To compare the relation of number of named work-related stressors and the stress level, we report Spearman rank correlations between the number of work-related stressors reported per day/week and the corresponding stress ratings (same day and weekly average), stratified by condition. Before all analyses, we conducted a paired Wilcoxon signed-rank test to examine whether participants’ average stress ratings differed significantly between Week 1 and Week 2. As no significant differences were found, all participants were included in subsequent analyses.

All statistical analyses were conducted in R (version 4.3.3; R Core Team) using nonparametric tests, as the assumption of normality was not met for most variables. A *P*<.05 was set as a criterion for significance. Missing data were analyzed as observed and not imputed.

### Coding of Open Answers

Voice messages were transcribed using Whisper Model (large-v2) [39] from speech to text before content analysis as a well-tested process for transcription [40]. Transcription was performed locally to maintain data privacy. For quality assurance, we manually checked 10% of transcripts. All open answers in the daily measurements about work-related stressors and energy sources were coded into categorical variables using 2 runs with different degrees of supervision. First, a Chat-GPT-o1 model deployed in Microsoft Azure OpenAI [41] was used to generate potential labels for both work-related stressors and energy sources loosely informed by the Job Demands–Resources model [42] with instructions to also consider non-job-related factors. The prompts used to generate these labels are provided in Multimedia Appendix 2. Coding runs were done separately for work-related stressors and energy sources and based on all open texts participants sent (ie, text and voice messages, plus additional text responses for extra stressors in the MC-condition). This resulted in a total of 311 different labels for work-related stressors and 582 labels for energy sources, as many were synonyms. We cross-checked results with 2 psychologists with expertise in the work health domain (JT and MBR). Based on the review of 10% of the material, labels were combined and redefined into 29 labels for work-related stressors and 31 labels for energy sources. The translation of the redefined labels, their definition, and examples are provided in Multimedia Appendix 3. In the second run, these newly defined labels were used, limiting output to the defined categories (classification task), including examples from the material. The prompts used for the second run are provided in Multimedia Appendix 2. All answers were manually checked for coding accuracy by one of the researchers (JT). The final classification accuracy was 95%.

Open answers in the browser-based evaluation questionnaire were categorized manually by a social psychologist (MBR).

### Power

To answer our research questions on feasibility, we needed to include 102 participants to detect a large effect between groups (d=0.5) with 80% power (based on a 2-sided independent *t* test). We set our recruitment goal to 250, assuming a conservative 50% dropout rate (defined as not starting or completing fewer than 80% of daily measurements) based on prior studies [19].

### Data Exclusion

All messages sent by participants who did not answer a question were deleted (eg, wishing the chatbot a nice day or telling it that they would be free the next day) as were answers given after midnight, creating a 6-hour window for response. Duplicate questions and answers, as well as day entries exceeding 10 days due to technical issues, were removed; only the first valid response was retained. Conversations with 17 people from wave 1 and 2 had to be removed as their telephone numbers could not be matched to an informed consent, likely because the QR code for sign-up was shared or scanned using a different device.

## Results

### Participants

**Figure 2.**
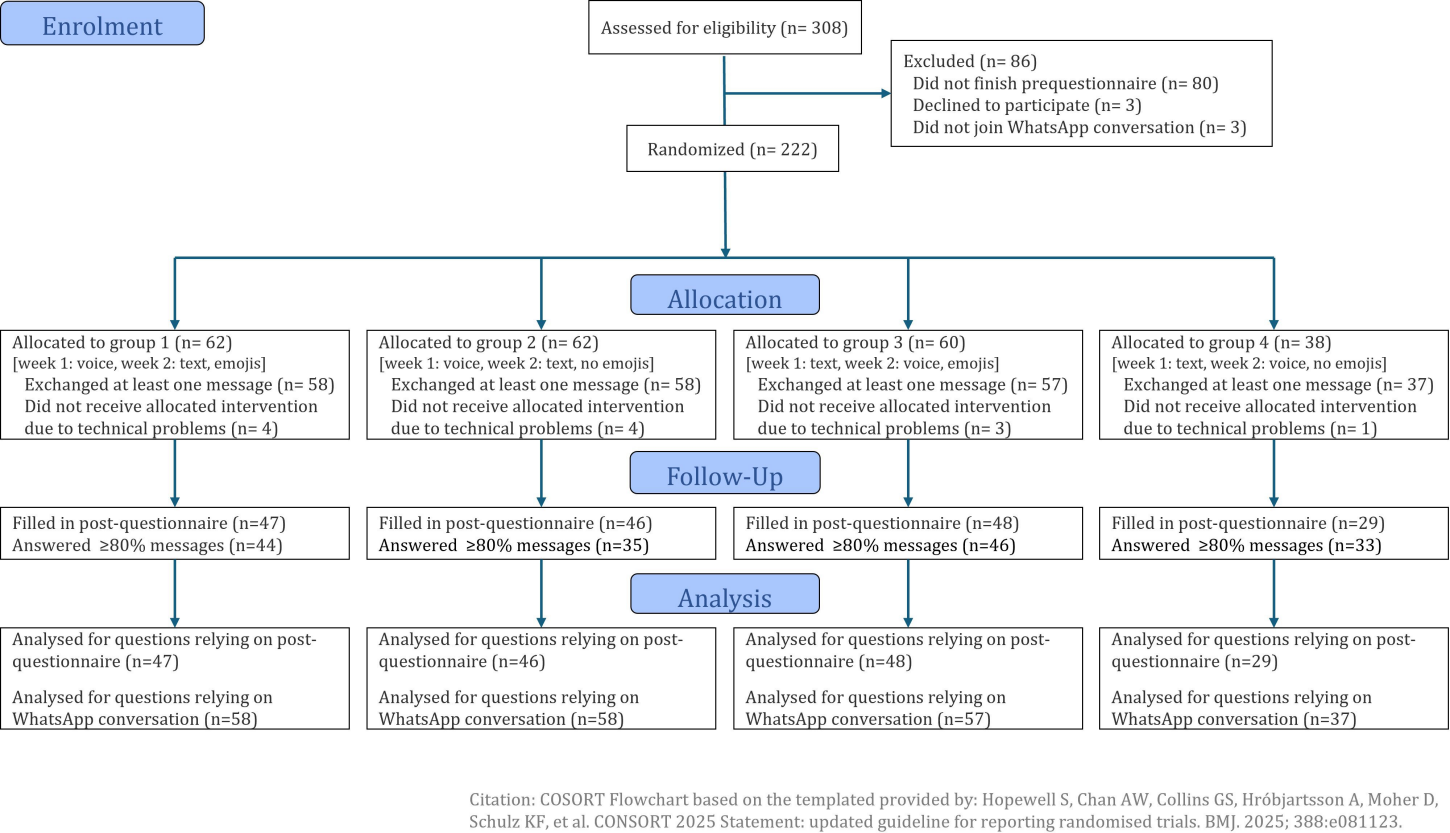
CONSORT (Consolidated Standards of Reporting Trials) flowchart of participants (adapted from Hopewell et al [43], which is published under Creative Commons Attribution 4.0 International License [34]).

In total, 222 participants gave informed consent and filled in the prequestionnaire. A total of 210 (95%) participants successfully joined the WhatsApp conversation and completed at least one daily measurement. Furthermore, 158 (71%) participants completed the daily measurements for at least 80% (adjusted for working days) and 170 (81%) participants completed the poststudy evaluation questionnaire (Figure 2). The sample was predominantly women (170/210, 81%), highly educated (173/210, 82%), and working part-time (109/210, 52%) (Table 1 displays the total sample and Multimedia Appendix 4 for group characteristics). The average stress level across the study period was 4.66 (SD 1.73) and did not differ between groups.

**Table 1.** Sample description based on prequestionnaire (N=210).

Variable and category	Values, n (%)
Gender	
Woman	170 (81)
Man	38 (18.1)
Other	2 (1)
Age (years)	
18 to 24	4 (1.9)
25 to 34	44 (21)
35 to 44	47 (22.4)
45 to 54	69 (32.9)
55 or older	46 (21.9)
Education	
Low	4 (1.9)
Medium	33 (15.7)
High	173 (82.4)
Workdays per week	
3 days	17 (8.2)
4 days	92 (44.2)
5 days	99 (47.6)
Use of voice messages in everyday life	
Never	34 (16.3)
Once in a while	73 (34.9)
Sometimes	70 (33.5)
Often	32 (15.3)

### RQ1: User Experience in Post Questionnaire

The postquestionnaire revealed a strong favor for the MC, as they were most frequently rated as more pleasant (75/170, 44%), easier (113/170, 67%), and safer (71/170, 42%) to use than the other methods, followed by text messages (Table 2). The open formats of text (57%) and voice messages (28%) were named more often regarding the method with which participants could better express themselves. As a general preference for research participation, WhatsApp questionnaires with MC questions (85/170, 52%) were rated highest, followed by traditional browser-based questionnaires (30/170, 18%). Technical problems were experienced by 15 participants (8.8%) with the voice messages and by 2 participants (1.2%) with both voice and MC questions.

**Table 2.** Usability rating in postquestionnaire (n=170).

Variable and modality	Total, n (%)
Nicer method	
Voice message	14 (8.2)
Text message	67 (39.4)
MC^a^ questions	75 (44.1)
No preference	14 (8.2)
Easier method	
Voice message	11 (6.5)
Text message	36 (21.2)
MC questions	113 (66.5)
No preference	10 (5.9)
Safer method	
Voice message	1 (0.6)
Text message	32 (18.8)
MC questions	71 (41.8)
No preference	66 (38.8)
Best to express oneself	
Voice message	48 (28.2)
Text message	96 (56.5)
MC questions	11 (6.5)
No preference	15 (8.8)
General preference for research method	
WhatsApp questionnaire (MC-questions)	85 (51.5)
Online questionnaire in a browser (like this one)	30 (18.2)
WhatsApp text messages	25 (15.2)
Other namely	9 (5.5)
WhatsApp voice message	8 (4.8)
(Telephone) call	4 (2.4)
Questionnaire in a special research app	3 (1.8)
Group discussion	1 (0.6)

a MC: multiple-choice.

The manually coded open answers to the question of what participants particularly liked or disliked about the different methods were consistent with the closed-question responses (Table 3). MC-questions yielded about 2.5 times more positive (n=138) than negative (n=55) aspects, with ease and convenience most frequently cited (n=102), although some participants noted the lack of nuance and context (n=30).

**Table 3.** Positive and negative aspects of the 3 question modalities as named in the postquestionnaire (n=170).

Modalities	Total
Voice messages	
Positive aspects	120
Ease of completion, speed, convenience	41
Ability to think aloud and organize thoughts	41
Ability to add nuance to answers, provide elaboration, offer context, suitable for long and complex answers	17
Ability to express emotions, describe feelings, share/convey feelings	12
Ability to use own words or articulate the story well	9
Negative aspects	167
Not suitable for every situation and time, uncomfortable with bystanders	74
Unpleasant or uncomfortable to send voice messages	29
Answers cannot be adjusted, must be correct in one go, little room/time to think about the answer, no room for reflection	21
Difficulty in articulating thoughts	19
Uncomfortable sharing voice data, uncomfortable sharing personal information in this way, does not feel anonymous	17
Feels impersonal, you do not know who you are talking to	4
Technical problems when recording/sending voice messages	3
Text messages	
Positive aspects	198
Space for elaboration, nuance, providing context	54
Easy, accustomed to text messages, convenient, quick	38
Time to think about the answer and possibly adjust it, offers a moment of reflection	38
Can be completed at any time, anywhere	34
Pleasant for concise answers (or unpleasant for long answers)	14
Feels anonymous/privacy feels ensured	10
Space for elaboration, nuance, providing context	10
Negative aspects	23
It takes time, it is difficult to formulate an answer	21
Other negative comments that do not fall into any of the other categories or are not further specified	2
Multiple-choice buttons	
Positive aspects	138
Easy, quick, requires little effort from the respondent, convenient	102
Providing options gives respondents guidance, opportunity to reflect, offers frameworks	28
Other positive comments not falling into any of the other categories or not specified	8
Negative aspects	55
No possibility for nuance, providing context, lack of depth	30
Answer options were missing, options were not comprehensive, cannot share personal experiences	24
Technical problems/objections	1

Text messages were predominantly evaluated positively, with 198 positive and 23 negative aspects reported. Participants highlighted the space for nuance and elaboration (n=54), ease of use and convenience (n=38), and time to think about the answer (n=38) as positive. A total of 21 participants mentioned that they found it difficult and time-intensive to formulate their answer in text messages.

Voice messages elicited nearly 1.5 times more negative aspects (n=167) than positive aspects (n=120). Positive aspects were similar to the ones mentioned about text messages and included ease of completion (n=41), time to reflect (n=41), and ability to provide nuance and context (n=17). Negative aspects particularly emphasized that voice messages were not suitable in all settings and that the timing of prompts was often inconvenient for recording voice messages (n=74). Twenty-nine participants reported feeling uncomfortable responding via voice messages.

### RQ2: Adherence

Mean adherence to the daily schedule was high (mean 95%, SD 33%). This is partly because 78 participants responded on more days than their typical working days, resulting in adherence rates of 100% or higher, even though adherence remains high after capping adherence rates at 100% (mean_capped_ 85%, SD_capped_ 23%). A screening of open responses suggests these participants may have had flexible schedules, as they mentioned working half days or completing tasks in peace. Mean adherence in week one was higher (mean 101%; mean_capped_ 90%) than in week 2 (mean 89%; mean_capped_ 79%). Participants tended to respond quickly to WhatsApp messages, with 50% of responses received within 13 minutes (mean 11.01 min, SD 427.79; 25th percentile: 0.35 min, Median: 13.33 min, 75th percentile: 69.38 min).

No statistically significant difference in adherence was detected between

the groups with (mean 96%, SD 36%) and without (mean 946%, SD 31%; W=5021, *P*=.43) emojis groups with the available data.

In week 1, a statistically significant difference was observed between the groups starting with the text condition (mean 107%, SD 24%) and in groups starting with the voice condition (mean 97%, SD 36%; W=6195, *P*=.046). No statistically significant difference was observed in week 2 (starting with text: mean 93%, SD 34%; starting with voice: mean 85%, SD 46%; W=5660, *P*=.48). The statistical test resulted in comparable results with the adherence rates capped to 100% (Multimedia Appendix 4).

### RQ3: Differences in Work-Related Stressors and Energy Sources Reported Per Modality

A total of 29 work-related stressors and 31 energy sources were identified in the open answers and used as categories to analyze the content of open responses from voice and text messages, as well as open-text answers following selection of the MC category “Other stressors.” Not all MC options were directly represented in the categories for open responses; however, most work-related stressors from the MC options had equivalents in the open-text categories, such as “Amount of work” and “Quantitative demands.” The open-response categories also included unique items not present in the MC options, such as “Understaffing,” “Health,” and “Travel or commute burden.”

The average number of work-related stressors per day chosen in the MC-condition and coded from the open answers given as voice messages differed significantly (W=13,917, *P*<.001). In the MC-condition participants reported an average of 3.32 (SD 2.15) work-related stressors per day compared with 1.3 (SD 0.96) work-related stressors named in voice messages.

**Figure 3.**
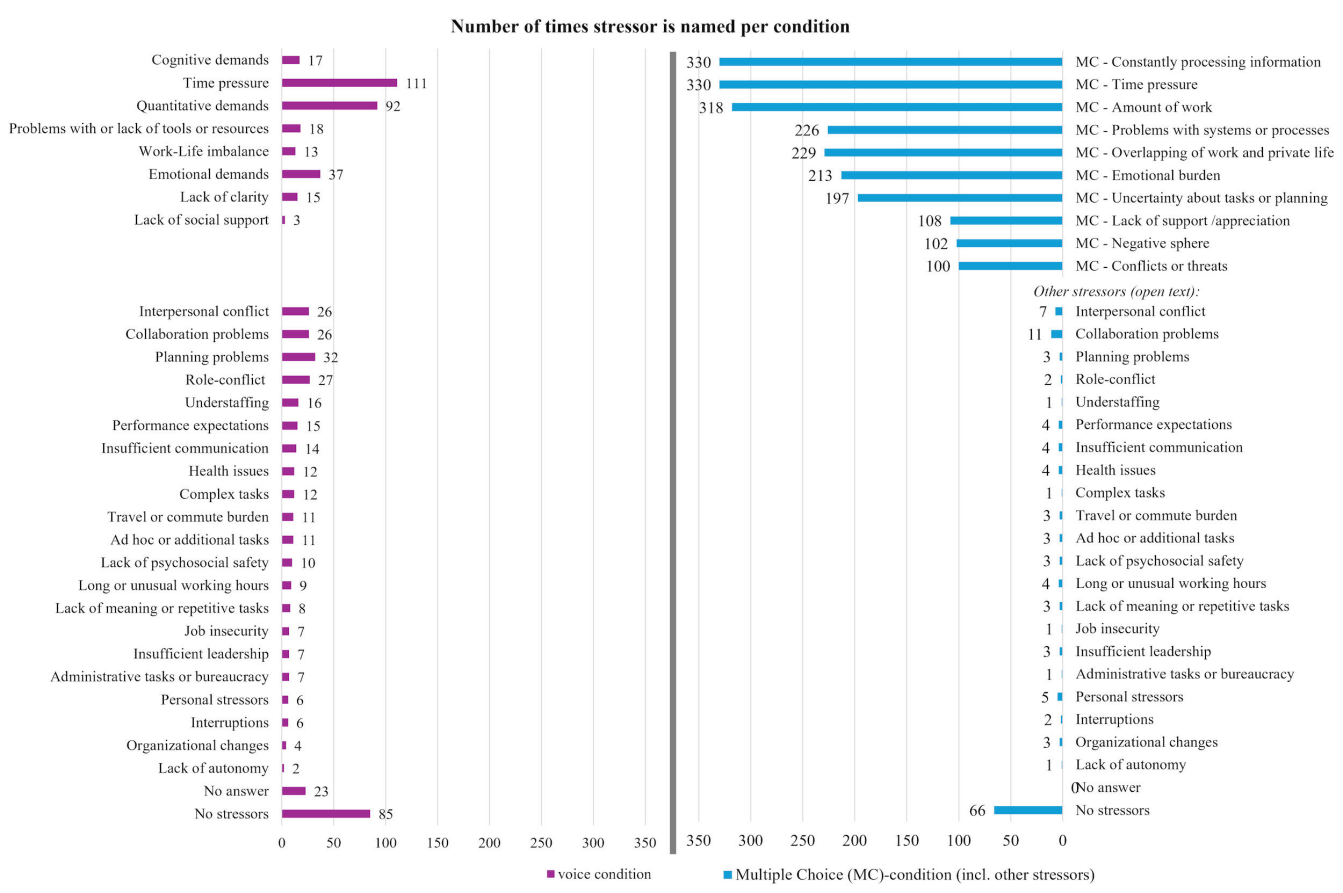
The number of total times work-related stressors were named in the voice or MC-condition, including open answers to the “other” category. MC-questions were not asked in the voice condition, and the closest category assigned to the open answers is presented alongside the MC-options. “Other stressors” described in open text were coded into the same categories as the voice answers. MC: multiple-choice.

The 2 conditions also differed in terms of which stressors were reported and their frequency. Figure 3 shows that MC options were selected between 100 times (“Conflicts or threats”) and 330 times (“Constantly having to process information”). In contrast, the most frequently reported stressor in the voice condition was “Time pressure,” mentioned 111 times.

The average number of energy sources per day reported in text messages and the number reported in voice messages did not differ significantly (voice: mean 1.65, SD 0.82 vs text: mean 1.55, SD 0.67; W=5231, *P*=.21). On average, participants used more words over the whole week to describe their energy sources in voice messages (mean 28.15, SD 23.48) than in text messages (mean 16.67, SD 8.92; W=10,197, *P*<.001).

**Figure 4.**
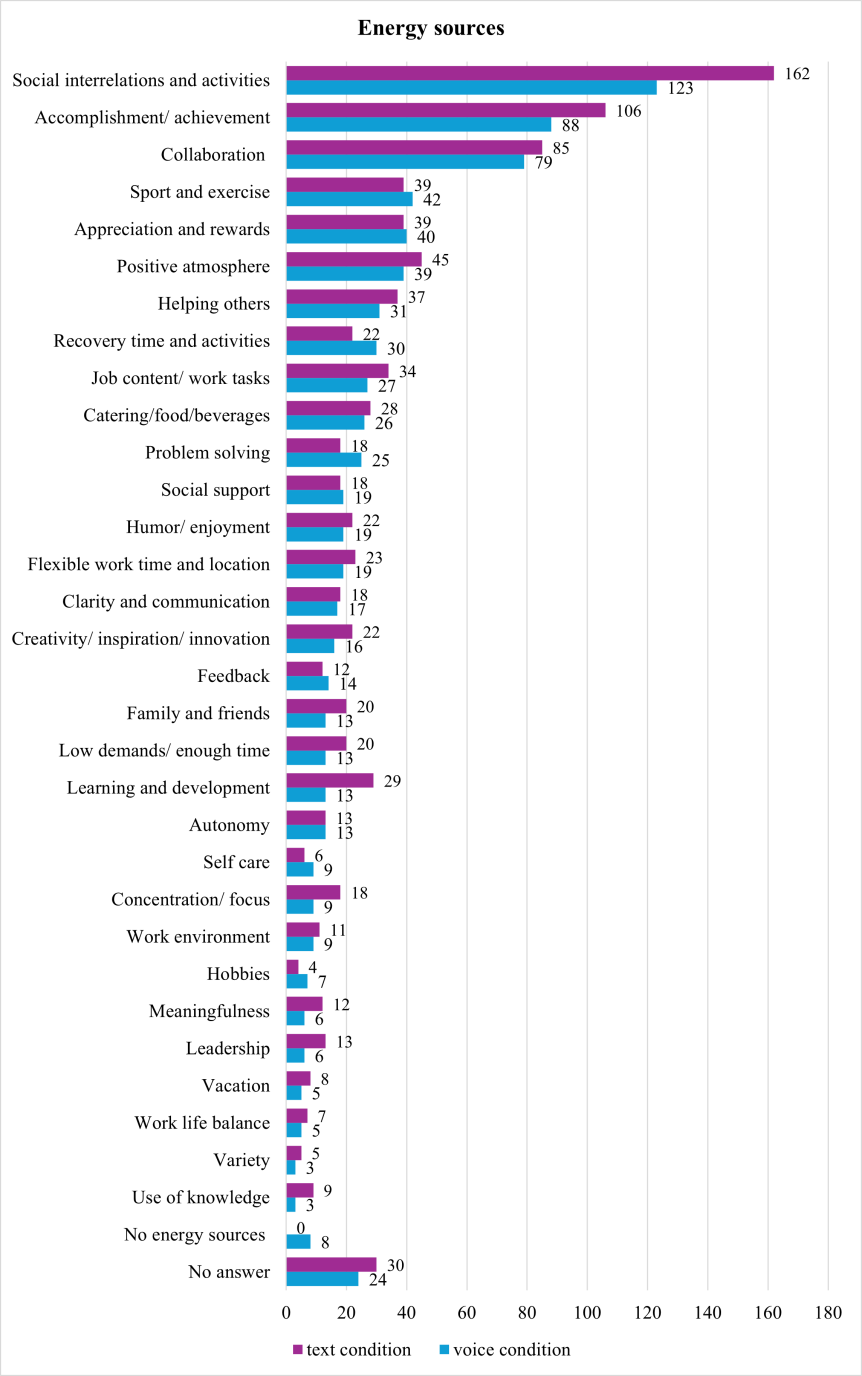
The number of total times energy sources were coded from the open answers in the voice and text conditions.

The types of energy sources (Figure 4) were similar in both conditions, with the same top 3 energy sources (“Social interrelations and activities,” “Accomplishment/ achievement” and “Collaboration”) in both conditions.

Considering the relation between the number of named work-related stressors on a daily as well as weekly basis with the responding stress level, the correlation was higher in the MC-condition. The number of MC stressors showed a stronger correlation at the daily level (*r*=0.58) and weekly level (*r*=0.49) compared with the number of stressors reported in the voice condition (daily: *r*=0.43; weekly: *r*=0.34).

## Discussion

### Principal Findings

The present study set out to explore the usability and feasibility of different methods for collecting daily stress experiences in the workplace through a semiautomated WhatsApp conversation. In terms of usability, participants (predominantly highly educated women) had a clear preference for MC-questions and text messages over voice messages as answer formats. Feasibility, in terms of adherence and data quality, can be considered good. However, differences in the number of reported work-related stressors and energy sources warrant cautious reflection on the suitability of these methods.

### User Experience

The results from the evaluation questionnaire indicate a strong preference of our sample for the MC-style questions with clickable buttons and text messages over voice messages. The main reasons cited were the convenience and ease of use in any situation, which is in line with general assumptions about question formats [44]. The biggest critique of voice messages was that not every situation was suitable for recording a voice message. This can be especially true in our study, as we had fixed-scheduled messages that could have occurred when participants were still in a work or commute environment, and the questions concerned that same environment. Personalizing the message schedule could therefore be advantageous. Crozier and Cassell [45] reported that participants perceived the flexibility of an audio diary as an advantage compared with a scheduled interview.

### Adherence

Overall adherence to the daily message (without limiting to usual working days) was high, with 95% of messages answered. Even when limiting adherence to the number of days usually worked (ie, adherence rate capped at 100%), adherence was 85%. This is exceptionally high, given that traditional diary studies are considered prone to missing entries [21]. In a recent systematic review [19], half of the EMA studies (defined as assessing well-being at least twice daily) reviewed reported daily completion rates averaging 72% (range 43%‐95%). The review noted that associations between adherence and age or gender were rarely reported, but higher completion rates were observed in shorter studies (1‐2 wk) [19,46]. In addition, responses to the initial message were typically quick (median 13 min), which is highly desirable for EMA studies [47]. The single daily measurement, short study duration, and restriction to working days in our study may explain the high adherence.

The use of emojis did not yield observable differences in adherence rates between groups. This is noteworthy given the vast research on emoji use in social marketing, which claims to enhance engagement [48,49] and to make chatbots more human-like [50,51]. However, this primarily relates to engagement on social media, such as “likes” and comments on specific posts or one’s own postings. In these contexts, the appeal of emojis is often attributed especially to women and younger generations [48,52].

Starting with the voice message method did, however, result in slightly lower response rates in the first week, in line with participants’ more negative evaluation of this method. Nevertheless, adherence in both groups was above 85%, even if capped at 100%. This may reflect both the selective sample and a possible novelty effect of the collection method. Our sample lacked sufficient diversity in age, gender, and adherence to examine subgroup differences, which is highly needed [19].

### Differences Between Modalities

Our results indicate a great difference in the average number of reported work-related stressors per day in the different conditions (MC-conditions: mean 3.32, SD 2.15; voice: mean 1.3, SD 0.96) as well as the different number of stressors named (10 MC-options vs 29 options derived from open answers). Literature on questionnaire methodology suggests that recall and familiarity bias can play a big role in how MC-questions are answered [44]. The absence of cues or examples in the open questions may partly explain this large difference. Thus, the question of whether to use open or closed questions persists, even within new technical solutions. This notion is long-standing, as Dohrenwend [53] in 1965 already suggested that in well-conducted questionnaires, open questions do not bear higher validity or information than closed ones, and the choice of question should always depend on the research question. Similarly, in a questionnaire on resilience and coping mechanisms, the authors found no added value of open questions over closed ones in predicting mental health outcomes [54]. Our finding that the number of MC stressors correlated more strongly with stress ratings than open-answer stressors points in the same direction within the context of diary-based stress measures.

All things considered, it is noteworthy that the open answers received contained a lot of information and were barely skipped, as is often observed in traditional questionnaires. This highlights the potential of open answers to collect more detailed insights on new topics [53].

The observable difference in the number of words used to report energy sources, which was higher in the voice condition, may reflect the unedited nature of spoken responses, as participants also suggested in the postquestionnaire. This aligns with prior research from Crozier and Cassell [45], which showed that in-depth audio diaries can yield additional insights not captured in “edited” written responses or closed questions, which can be interesting for studying thought and reasoning processes. As diary and EMA studies—besides data collection—are also often reported to give insights and offer reflection moments, greater attention to the reflection process appears warranted [21]. In the evaluation questionnaire, both open-ended formats were mentioned to aid reflection, and voice messages were particularly described as helpful for “organising one’s thoughts.” Indeed, a study on teacher reflections found that written reflections were more summative, whereas spoken reflections provided greater detail and reasoning [55]. In the context of measuring stress, reflecting on it and potentially acting on it, these findings highlight the need for a deeper understanding of these differences in modalities.

### Implications

The differences in assessed work-related stressors when using different methods support Crozier and Cassell’s [45] call for pluralism in stress research. In the following section, we discuss situations and research aims for which the methods explored in this study may provide added value. Starting with participants’ preferred method, the MC question, this format most closely resembles a traditional questionnaire, albeit with a different technical implementation. The evaluation survey showed a preference for WhatsApp questionnaires with buttons (MC-condition) from half the sample, followed by a traditional (browser-based) questionnaire and open-text questions in WhatsApp.

As the comparison to the open-answer question showed, participants reported on way more different stressors than we suggested with the MC options. MC questions are therefore most suitable when the symptoms or behaviors of interest are already well defined. To ensure relevant answer options, we recommend a participatory approach in which MC options are derived from the target group (eg, the most common stressor for a certain profession or type of work). Research questions then could center around incidence or accumulating effects of those stressors. To leave some more room for personalization and nuance, an alternative could be adaptive MC-questions, where next to predefined answers, participants can create their own answers, which are stored and shown to them in the following measurements as possible in specialized EMA apps [56]. However, this and other question types (eg, ranking or matrix questions) require technology beyond the consumer-ready marketing platforms currently available for social media.

The open text messages were also still conceived as convenient and quick for participants in the evaluation questionnaire. Interestingly, participants still occasionally (n=21) described them as time-intensive in the postquestionnaire, a negative aspect not raised with the voice messages. Participants acknowledged that it was easier to describe, in both open answer formats, nuanced and in context, what they experienced that day. While closed MC-questions can create a quick picture of the frequency of, for example, different work-related stressors and energy sources, open questions may play a crucial role in informing answer options, particularly for novel topics [53] and in revealing contextual information that influences measurement. If we look at the amount of different work-related stressors and energy sources, we find a large variety, including work-related energy sources such as autonomy, social support, and feedback [57] and energy sources outside of work such as hobbies, time spent with family, and recovery, indicating the open questions to be a rich information source. Insights from open responses illustrate concrete situations, offering opportunities to develop personalized advice for individuals and employers.

The general willingness to respond in written form within a conversational-style assessment suggests promise for developing stress assessments based on conversational agents rather than static chatbots, which is a rapidly emerging field. As participants increasingly recognize the conversational benefits of LLMs such as ChatGPT, the ability to systematically assess information comparable to questionnaires presents an interesting avenue for future research [58-60]. Additionally, written electronic diary entries combined with LLMs open up new therapeutic approaches that were not feasible before the advent of these models [61]. In this regard, our study demonstrates how LLMs can be used to analyze written material efficiently and semiautomatically, based on scientific models, thereby reducing barriers to incorporating open responses in stress research.

Our study does not support the idea that giving the option to answer with voice messages increased participation; it merely did not seem to hinder it in the current sample. As a standalone collection method, voice messages cannot be recommended unless explicitly designed with and for a specific target group (eg, younger individuals, people with visual impairments, or those with language difficulties [21]; . Given the comparable content, one could give participants the option to choose whether they answer per text or voice message. However, this option is already available on most smartphones via a dictation feature. Specific research questions may still require voice recordings, in which case WhatsApp or other messaging services could provide a feasible collection method. For example, research using voice recordings to diagnose and monitor health and disease [62] could facilitate audio recordings or combine them with questionnaire items or other biomarkers, thereby minimizing patient burden.

### Limitations

Several limitations of this exploratory usability and feasibility study should be considered when interpreting the results. First, despite the envisioned low-threshold and inclusivity of WhatsApp as a research tool, our sample predominantly consisted of highly educated women, a demographic overrepresentation that is common in this type of research, as a systematic review revealed that more than half of EMA studies reported an overrepresentation of women and highly educated persons [19]. This imbalance was likely amplified by our recruitment strategy, which included outreach through scientific networks and opaque advertisement algorithms on social media platforms. Future studies should aim to diversify sampling strategies to ensure broader generalizability of the findings.

Second, while our study used a novel method for assessing stress, we did not compare this approach with established questionnaire-based assessments, nor did we validate the EMA items. This lack of methodological triangulation limits the evaluation of convergent validity and may affect interpretation of the measured constructs. This is a logical next step after proving the general feasibility of this collection method with this study.

Third, we used a single daily measurement, aligning our design more closely with traditional diary studies than with EMA protocols involving multiple daily prompts. End-of-day diary entries and EMA are not fully interchangeable, although they tend to yield more comparable results than weekly stress ratings [63]. This nuance may influence the granularity and ecological validity of the findings in our study. However, given that the overall burden was perceived as low in the evaluation questionnaire, this approach is likely also feasible with multiple prompts per day.

Fourth, logistical limitations in the technical setup may have impacted participant engagement. Participants could not begin automated data collection immediately after enrollment, as group assignment and message scheduling were handled manually. This could result in delays of up to one week, potentially reducing initial motivation. Additionally, we used 2 different data collection platforms—Survalyzer for informed consent and WhatsApp for the actual daily measures—due to ethical concerns of asking for consent via WhatsApp. Although no direct impact on participation was observed, this switch may have introduced variability in user experience and warrants refinement in future implementations. As assessments and feedback mechanisms become more complex, new technological challenges are likely to emerge.

Last, our approach to coding open-ended responses using LLMs is innovative but lacks established validation within psychological research. To mitigate this, we monitored outputs carefully, always manually double-checked 10% of responses at each stage with experienced psychologists and fully verified final categories before analysis, achieving high agreement between LLM coding and human coders. Nevertheless, the use of LLMs remains an emerging practice that requires further validation.

### Conclusions

Conducting diary studies via WhatsApp as a convenient, low-threshold method was perceived positively by participants, with a preference for MC questions and text messages over voice messages, due to their convenience and suitability for responding in any situation. However, our relatively homogeneous sample of mostly highly educated women leaves open the question of whether results would differ in more diverse samples. The high adherence, richness, and completeness of information gained through the study render this method feasible and promising for future-proof (work) stress research, provided it is carefully aligned with study goals and validated through direct comparison with traditional methods.

## Supplementary material

10.2196/84032 Multimedia Appendix 1 Screenshots of conversation with the chatbot in both conditions (with emojis).

10.2196/84032 Multimedia Appendix 2 Prompts used for unsupervised coding of open answers about work-related stressors and energy sources.

10.2196/84032 Multimedia Appendix 3 Final categories used to code open answers on energy sources and stressors.

10.2196/84032 Multimedia Appendix 4 Sample description based split by groups and adherence rates when capped at 100%.

10.2196/84032 Multimedia Appendix 5 Copilot conversations.

## Abbreviations

EMA: ecological momentary assessment
LLM: large language model
MC: multiple-choice
TNO: The Nederlands Organization for Applied Scientific Research
