# Supplementary material for: Using Semiautomated WhatsApp Messages for Daily Stress Measurements: Integrated Usability and Feasibility Study
Source: JMIR Form Res. 2026 Mar 11;10:e84032. doi: 10.2196/84032 (PMC12978546; doi:10.2196/84032)
Supplement: Multimedia Appendix 1 [file formative-v10-e84032-s001.pdf]

Multimedia Appendix 1 – Screenshots WhatsApp chatbot

Voice condition (with emojis):

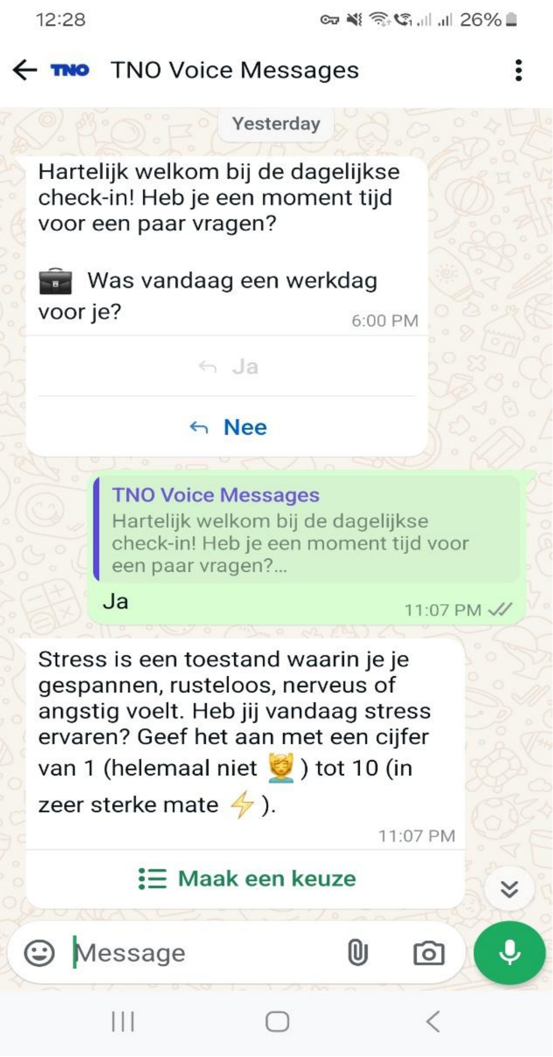

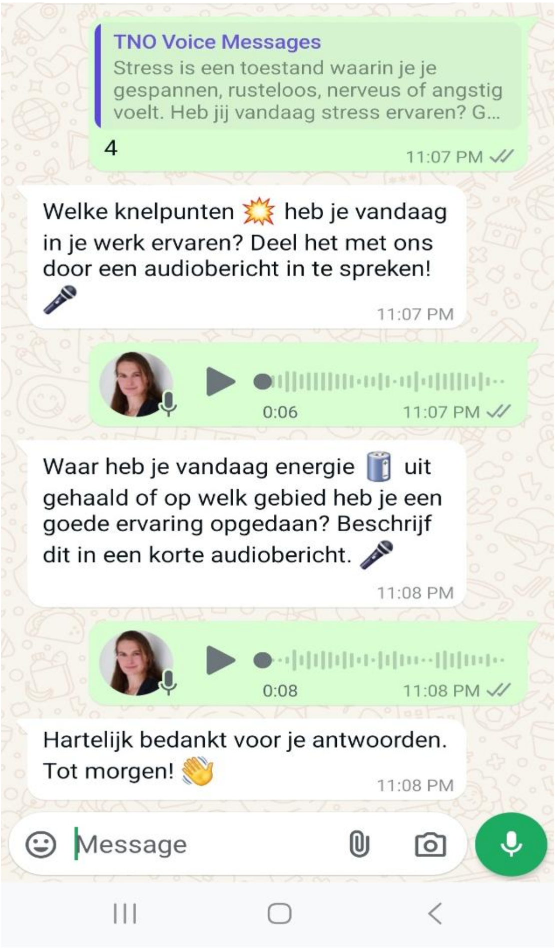

This multimedia appendix belongs to: Thielecke et al. (2026). Using Semiautomated WhatsApp Messages for Daily Stress Measurements: Integrated Usability and Feasibility Study

Text-/ MC-condition (with emojis):

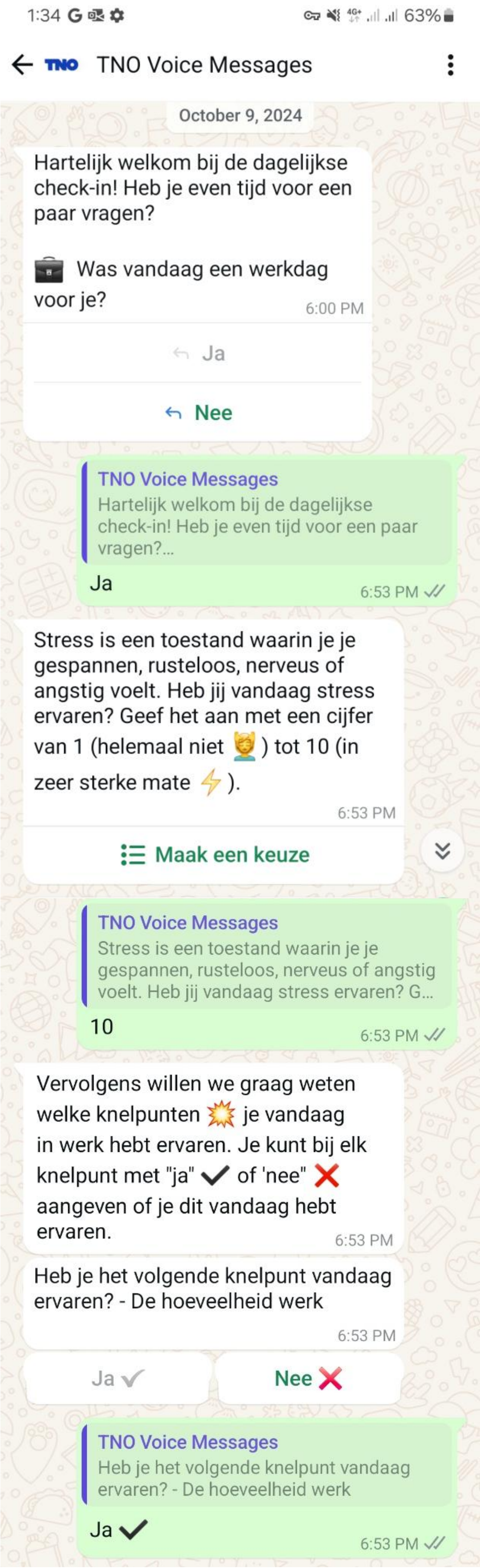

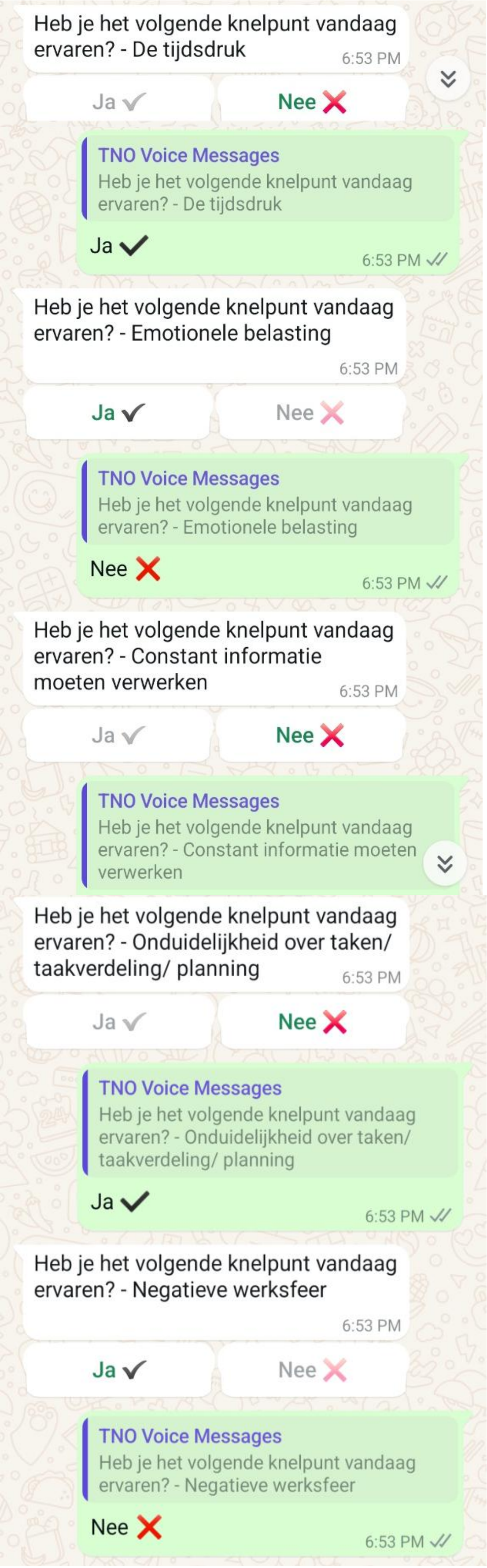

This multimedia appendix belongs to: Thielecke et al. (2026). Using Semiautomated WhatsApp Messages for Daily Stress Measurements: Integrated Usability and Feasibility Study

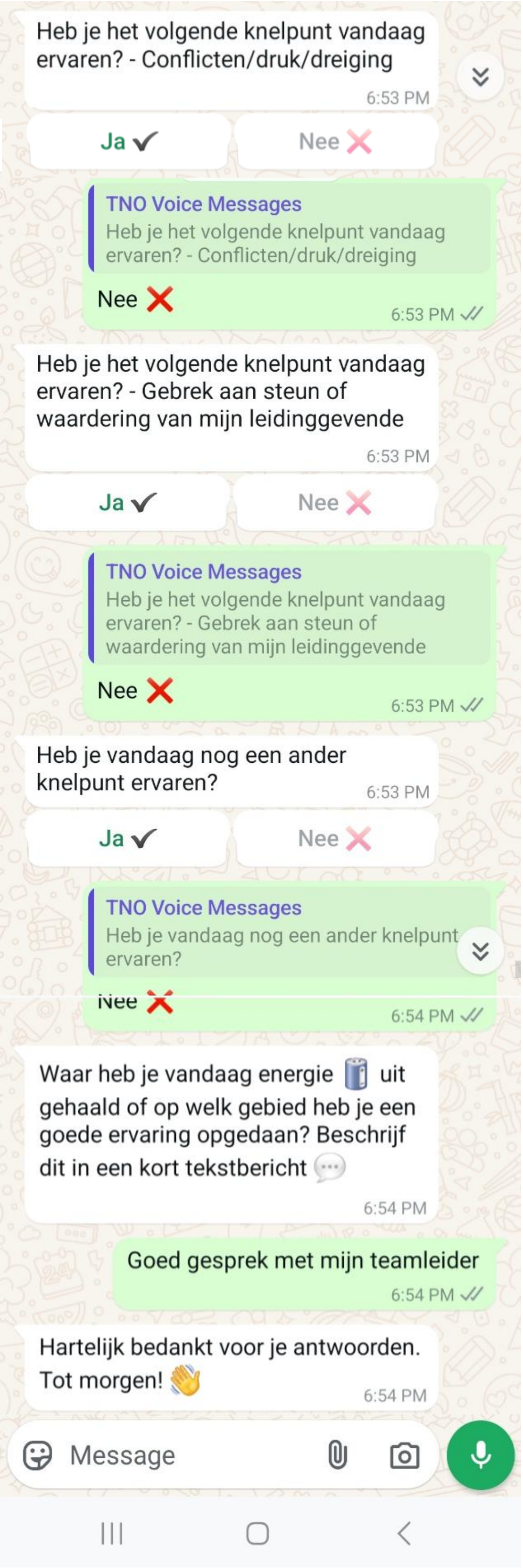

This multimedia appendix belongs to: Thielecke et al. (2026). Using Semiautomated WhatsApp Messages for Daily Stress Measurements: Integrated Usability and Feasibility Study
