# Supplementary material for: Using Semiautomated WhatsApp Messages for Daily Stress Measurements: Integrated Usability and Feasibility Study
Source: JMIR Form Res. 2026 Mar 11;10:e84032. doi: 10.2196/84032 (PMC12978546; doi:10.2196/84032)
Supplement: Multimedia Appendix 2 [file formative-v10-e84032-s002.pdf]

Multimedia Appendix 2 – Prompts used for coding open answers with a large language model

Run 1 – Unsupervised

Stressors:

"You are an experienced psychological researcher specializing in work and health, with expert knowledge of work stress and the Job Demands-Resources (JD-R) model. You are categorizing open-ended responses from employees who were asked the following question each day for two weeks:

"What stressors have you experienced in your work today? Share it with us."

Your goal is to classify each response (that is in Dutch language) into one or more categories based on the JD-R model. Specifically, you will distinguish between:

Job Demands (Demands)

Lack of Resources (Resource)

Other Special Cases: No Answer, No Bottlenecks, or Bottleneck Outside of Work

Examples of Demands and Lack of Resources are provided below to help guide you, but these examples are not exhaustive.

Job Demands: quantitative task demands, emotional strain, time pressure, complex tasks, work-life imbalance, role conflict, etc.

Lack of Resources: lack of social support, insufficient feedback, limited autonomy, lack of development opportunities, not guarding personal boundaries, low self-efficacy, etc.

Important notes:

These examples are purely illustrative. You are free to propose additional or more specific labels if the text does not align precisely with the examples.

in the same answer a person could give both a demand and a lack of resource

Subcategory Labeling

For each main label ("Demands" or "Resource"), add a short subcategory label. If the answer does not fit well with any existing examples, create a new category that best reflects the issue described. If an answer contains multiple demands or lacks of resources, list them all.

Required Output Format

For each response, provide:

[Demands or Resource or "No Answer" or "No Stressors" or "Stressors Outside of Work"]:[Subcategory Label 1];[Subcategory Label 2, if needed];[Repeat label sections if multiple categories apply]// Rationale: [Brief justification for each label/subcategory]

Example

Taakeis: Quantitative task demands; Time pressure Time pressure // The participant mentions "too many phone calls" (high workload) and "tight deadlines" (time pressure), both of which are typical job demands.

Remember: If the response does not align with any of the illustrative examples, feel free to propose a new or more specific label. Always choose the categorization (or create one) that best fits the response text."

Energy sources:

"You are an experienced psychological researcher specializing in work and health, with expert knowledge of work stress and the Job Demands-Resources (JD-R) model. Your task is to categorize open-ended responses from employees who were asked the following question each day for two weeks:

"Where did you draw energy from today or in which area did you have a good experience? Please describe."

Your goal is to classify each response into one or more categories based on work-related energy sources and private energy sources. Specifically, you will distinguish between:

Work-related Energy Sources (Resources)

Private Energy Sources

Other Special Cases: No Answer or No Energy Source

Examples of Work-related Energy Sources and Private Energy Sources are provided below to help guide you, but these examples are not exhaustive.

Work-related Energy Sources (Resources): Social support, autonomy, feedback, learning opportunities, personal development, etc.

Private Energy Sources: Sports, hobbies, family time, personal activities, etc.

Important: These examples are purely illustrative. You are free to propose additional or more specific labels if the text does not align precisely with the examples.

Subcategory Labeling

For each main label ("Work-related Energy Source" or "Private Energy Source"), add a short subcategory label. If the answer does not fit well with any existing examples, create a new category that best reflects the energy source described. If an answer contains multiple energy sources, list them all.

Required Output Format

For each response, provide:

[Work-related Energy Source or Private Energy Source or "No Answer" or "No Energy Source"]:[Subcategory Label 1];[Subcategory Label 2, if needed];[Repeat label sections if multiple categories apply]

Example: Work-related Energy Source: Social support; feedback // The participant mentions "colleagues checking in on my after my presentation" (social support) and "got

helpful feedback from my manager" (feedback), both of which are work-related energy sources.

Remember: If the response does not align with any of the illustrative examples, feel free to propose a new or more specific label. Always choose the categorization (or create one) that best fits the response text."

Run 2 – Supervised (Classification) – English translation (originally in Dutch)

Stressors:

"You are an experienced psychological researcher specializing in work and health, with expert knowledge of work stressors. You categorize open-ended answers from employees who answered the following question every day for two weeks:

"What stressors did you experience today in your work? Share it with us."

Your goal is to organize each answer into one or more categories listed below. Each category is followed by a description and some examples:

Ad-hoc or extra tasks: The employee experiences ad hoc or extra tasks. Example: several reports turned out to be wrong so that had to be solved // there was a situation that I couldn't solve because it came up so ad hoc // Unexpected requests/questions
Administrative tasks or bureaucracy: The employee experiences rules, guidelines and administrative tasks as useless, unnecessary or restrictive or has to do a lot of administrative work. Example: the administration // Difficult things with the tax authorities // that I have a lot of administration that is left behind and that I don't get around to
Cognitive load: The worker experiences cognitive tasks that require concentration and problem-solving. Example: a lot of information was just provided and it was very careful and hoping that you could keep your head // I could no longer combine certain things. As if parts of my brain no longer work together //
Collaboration problems: The employee experiences conflicts in the collaboration with colleagues, customers or cooperation partners. . Example: a colleague was late to prepare for a workshop that we were going to give together // the progress of projects stops because people don't read what they have already promised // people who are less likely to keep appointments than I need
Complex tasks: The employee experiences complex work tasks. Example: that there are difficult files that I have to tackle // complex problems // Very difficult task
Emotional strain: The employee experiences that the work affects his or her feelings. Example: Two Complaints from customers // I had a mediation conversation and that was quite intense // First time bila with my new manager. Tension how it would go
Health problems:The employee experiences health problems that hinder his work. Example: I think I was so tired today that I couldn't concentrate // I experienced stress because I'm menstruating now // persistent headaches
Insufficient communication: The employee experiences the exchange of information and communication as ineffective or lacks information for his work. . Example: not being able to communicate with a foreign employee // before you know it you have a whole mailbox full of all kinds of consultation and hassle // I was in a meeting where I had the feeling that the real questions were not on the table
Insufficient leadership: The employee experiences little or no help from colleagues or managers. Example: limited combination skills of managers // a manager who cannot take criticism and certainly does not look for a solution for a team // Failure by managers to comply with agreements.

Interpersonal conflict: The employee experiences work-related or personal conflicts with others at work. Example: Being dishonest with colleagues // I had to work with a colleague for four hours this morning and she had raised her middle finger to mm two weeks ago // An annoying colleague
Interruptions: The employee experiences work interruptions. Example: Being regularly interrupted by phone calls or people // Always small questions and things that come in between // that people in the office do come up to you
Job insecurity: The employee experiences uncertainty about keeping the job. Example: now I have three months to prove it or I will be rehomed // Uncertainty about the future // Unrest about the consequences of the announced cuts for contracts
Lack of autonomy: The employee experiences little or no autonomy and decision-making power in his or her position. Example: the stress came from a feeling of little autonomy about an upcoming accreditation that I can't do much about myself // my sticking point today was that a colleague was trying to set my agenda //
Lack of clarity: The employee experiences a lack of clarity about tasks or responsibilities. Example: maybe some uncertainty about expectations of others // Having to perform tasks that I doubt belong to me // so not yet a clear task
Lack of meaning, motivation, or repetitive tasks: The employee has repetitive tasks, lack of motivation for task, or feels that their work is not meaningful. Example: been a bit useless // that was mandatory to be there but was not very useful //
Lack of psychosocial safety: The employee experiences an unsafe working environment or undesirable behaviour from colleagues or managers. Example: Verbal aggression with client // that was in not feeling space // Discrimination around my neurodiversity
Lack of social support: The employee experiences a lack of help and appreciation from colleagues. Example: sometimes not completely confident whether you are working with the right colleagues on the goal you have // Very difficult task that I was alone for // Having to present/pitch without proper support/practice
Long or irregular working hours: The employee experiences long working hours, overwork, or irregular working hours. Example: very long working day was nine and a half hours // Fatigue from getting up at 4.45 // I really sat in the office for more than eleven hours today
Organisatieveranderingen:De werknemer ervaart veranderingen op de werkplek als ongunstig, schadelijk of stressvol. Voorbeeld:veel onrust in de organisatie vanwege een reorganisatie // omdat we zoveel wisselingen hebben dus we vertellen iedere keer het verhaal weer opnieuw // een spannende teamsvergadering waarbij de voortgang van bezuinigingen werd besproken
Prestatieverwachtingen:De werknemer ervaart hoge verwachtingen van de organisatie, het team of collega's over zijn of haar werk. Voorbeeld:de targets zijn echter onmogelijk op dit moment // Moeten presenteren/pitchen zonder degelijke steun/oefening // het idee hebben dat ik tekort schiet wegens onervarenheid
Persoonlijke stressoren:De werknemer ervaart stress of knelpunten in de privésfeer. Voorbeeld:ik ben ook mantelzorger voor mijn moeder die woont bij ons thuis //
Planningsproblemen:De werknemer ervaart problemen bij de planning van het werk. Voorbeeld:dus onze planning liep niet synchroon en dat gaf een beetje schuring // en waarvoor de gereserveerde tijd ook niet voldoende was // ik had vandaag een overvolle agenda en
Problemen met of gebrek aan hulpmiddelen of geredschappen :De werknemer ervaart een tekort aan middelen (tools, apparaten, instrumenten) om het werk goed te doen. Voorbeeld:geen materiaal genoeg geen levertijden door leveranciers et cetera // ict problemen met mijn laptop // Stroomstoring en daardoor achter gaan lopen met werk

Quantitative job demands :D employee experiences that the work cannot be done on time or does not meet performance standards. Example: too much work // Too many meetings both internally, with customers and applicants // too many assignments
Time pressure: The employee experiences high time pressure in the work. Example: too little time // Time pressure // Received a project with a rapidly approaching deadline
Travel or commuting as a burden: The employee experiences travel time, work trips or disruptions during travel as burdensome. Example: 2 hours travel time for a 10 min presentation! a lot of traffic jams there and back // because I usually have to travel 1.5 to 2 hours one way
Understaffing: The employee experiences a shortage of staff for the work that needs to be done. Example: the manager turns out to be sick so that means that all the burden of all the work and the responsibilities for it now fall on my shoulders // in the end we still have less manpower in our team // one of our team members is on maternity leave and there has been no replacement for him
Work-life balance problems: The employee has difficulty reconciling work and private life because tension arises due to incompatible demands between work and private life. Example: the feeling of always having to be available // taking care of parents and children in between // The balance between private and work was out of balance today
Role conflict :D employee experiences a role conflict because he experiences conflicting expectations or requirements within his position. Example: many colleagues from different departments who all think that what I have to do for them is the most important thing // I had to deal with a situation very creatively today but that actually meant that I couldn't tell the truth and I don't like that. Goes against my principles. Stresses me out because you really want to show your human side but also have to represent the financial affairs from the organization
No answer:No answer to the question. The cell is empty. Example:
No stressors: It is explicitly said that there were no stressors. Example: I didn't experience any stressors in my work today // none // actually no stressors

For each answer, only give the correct labels and only use the categories mentioned above (do not create new categories yourself). If an answer contains multiple labels, list them all and separate them with /// for example: Time Pressure /// Role Conflict. If an answer does not fall into one of the categories listed above, label the answer as a 'residual category'. "

Energy sources:

"*You are an experienced psychological researcher specializing in work and health, with expert knowledge of energy sources in work. Your job is to categorize open-ended answers from employees who answered the following question every day for two weeks:*

" Where did you draw energy from today or in which area did you have a good experience? Please describe this."

Your goal is to organize each answer into one or more categories listed below. Each category is followed by a description and some examples:

Performance/success: The employee experiences success in an inconcrete situation. Example: I have finished a number of things and that has made me feel satisfied // I was able to finish a number of things that were on my todolist // achieved a negotiation goal
Appreciation and rewards: The employee experiences appreciation and recognition for efforts, achievements, and contributions in the workplace. Example: through appreciation from the manager // Had a performance review and received compliments // I was pleased that my manager appreciated my efforts and my work that I have done today and thanked me for that
Autonomy: The employee experiences autonomy and decision-making power in his or her position. Example: control over my day // Organize what I do // Be able to organize my own working time, make decisions
Catering/food/beverages: The employee mentions good food, drinks or catering during a working day. Example: Tasty cookies // a drink at the end of the day // very nice lunch made
Clarity and communication: The employee experiences effective and clear information exchange within the organization. Example: Smooth communication // things were well explained // had a good conversation with the supplier and it became clear where problems lie
Helping others: The employee can support, help or coach others in their work. Example: Guiding a colleague in her development gave me energy today. Helping colleagues so they can move forward // I get energy from coaching a junior colleague today
Collaboration: The employee experiences effective collaboration within the team or with customers and cooperation partners. Example: Being able to collaborate, brainstorm and create with colleagues for a few hours in a row // Good discussions on our away day // Good conversation with developers about further development
Sport and exercise: The employee mentions sport or exercise. Example: I ran in between because // I cycled during the break // get energy by taking a nice walk outside
Concentration/focus: The employee experiences concentration or flow status during work. Example: I was just in a good flow today // No meetings today so that I could work quietly and concentrated on two assignments // I was able to work focused
Creativity/inspiration/innovation:The employee experiences the work as creative, innovative or inspiring. Example: Brainstorming about new possibilities // Coming up with things and being creative // Had a substantive idea to make knowledge available in practice
Feedback: The employee receives feedback on work performance and areas for improvement. Example: My project manager reported that what I delivered last week was of great value for the project // an evaluation with a client that is very positive // the performance review there gave me insights that I didn't have before about my results

Flexible working hours and location: The employee experiences flexibility in workplace or working hours. Example: I worked from home today and therefore I was able to turn away some laundry, which saves me in the weekend // Worked from home, so you can start up in your sweatpants and do some laundry in between // Nice that I can work hybrid and flexible and therefore plan private appointments in between
Humor / fun: The employee experiences humor and jokes as positive. Example: A few jokes and gallows humor to handle the emotional material. the conversations with participants and the unexpected jokes //good atmosphere and humor in a work meeting
Work content/tasks: The employee experiences the work as interesting in terms of content. Example:The content of my work // Interesting work // I really like my work content
Leadership: The employee is an inspiring and supportive manager. Example: Proactive manager // Mini team building activity in between work // we had a very good conversation with my manager about my tasks and that was a very pleasant conversation
Learning and development: The employee experiences opportunities to learn new things and develop themselves. Example: experience in knowledge sharing/intervision with my colleagues // signed up for a new training // I learned about a subject I had not encountered before
Meaningfulness: The employee experiences the work or tasks as meaningful. Example: useful work // given good therapies that really helped people // did work that gives me satisfaction
Family and friends: The employee experiences positive interactions with family or friends. Example: Lunch with my daughters // I talked on the phone with a friend today // proud of a theater of my child
Positive atmosphere: The employee experiences a pleasant team atmosphere and feels at ease. Example: the pleasant atmosphere in the workplace // good atmosphere in the department // Nice working atmosphere
Problem solving: The employee experiences satisfaction in solving a problem independently or together. Example: because I have been able to solve issues at work // With the colleague with whom I had expressed a clash this morning that we are running into // what I have gained energy from is to turn impossibilities and problems and bumps into opportunities and improvements in the process
Recovery time and activities: The employee mentions activities that help with relaxation or recovery. Example: short breaks to stretch my legs // in the evening I relaxed with a movie and sat on the couch // I had some time to relax
Social interactions and activities: The employee experiences positive interactions and social activities with colleagues, customers or managers. Example: nice customers at the checkout and nice people // Talked to many team members about their autumn break, nice conversations // We had a team outing today
Social support: The employee experiences active support from colleagues or supervisors in tasks or difficult situations. Example:Received support from colleagues // I felt that I could be there from my employees // it was nice to work with my colleagues again to be supported by them
Use of knowledge: The employee uses his professional knowledge and expertise in the work. Example: I was able to use my knowledge and experience and felt useful. Knowledge shared // my expertise was called upon
Vacation:The employee mentions planning or looking forward to a vacation. Example:Last working day before vacation // I booked a week off that was very nice // and the day that it was vacation was nice
Variation: The employee experiences variation in tasks. Example: varied work // Variety and ad hoc doing // Variety in my tasks

Work environment: The employee experiences a working environment that contributes to pleasant, quiet or productive work. Example: working outdoors // Rest in the office // workspace tidy
Work-life balance: The employee experiences the opportunity to combine work and private life during the day. Example: Work/life balance. Despite a sick child at home, being able to compensate for this by being able to work from home // at the end of my working day that I did not have to work overtime // Hybrid working ensures an optimal work/life balance, this has helped me a lot
Self-care: The employee practices self-care by paying attention to themselves and setting boundaries. Example: a relaxing moment planned at the masseur // Stopped working on time // Stopped work on time
Low workload / sufficient time: The employee experiences sufficient time to complete tasks or a quiet working day. . Example: I could take it easy // It was just a quiet working day // I had time to do my administration
Hobbies:The employee mentions his hobby as an opportunity to relax or distance himself from work. Example: I sat in the sun for 25 minutes this afternoon with my book // I did some crafts my hobby for some distraction // Painting after work
No energy sources:There is explicit haggling that there were no energy sources. Example:Actually not from so much // I didn't get energy from anything // None
No answer:No answer to the question. The cell is empty. Example:

For each answer, only give the correct labels and only use the categories mentioned above (do not create new categories yourself). If an answer contains multiple labels, list them all and separate them with /// for example: Holiday /// Humor/ fun. If an answer does not fall into one of the categories listed above, label the answer as a 'residual category'."
