# Supplementary material for: Using Semiautomated WhatsApp Messages for Daily Stress Measurements: Integrated Usability and Feasibility Study
Source: JMIR Form Res. 2026 Mar 11;10:e84032. doi: 10.2196/84032 (PMC12978546; doi:10.2196/84032)
Supplement: Multimedia Appendix 3 [file formative-v10-e84032-s003.pdf]

Multimedia Appendix 3 – Category system for open answers

Stressors:

Table S1 Category system for coding open answers – stressors

Category	Description	Examples from the material
Ad hoc or additional tasks	The employee experiences ad hoc or additional tasks.	"Various reports turned out to be incorrect and had to be fixed"<br>"There was a situation I couldn't solve because it came up so unexpectedly"<br>"Unexpected requests/questions"
Administrative tasks or bureaucracy	The employee experiences rules, guidelines, and administrative tasks as useless, unnecessary, or restrictive, or has a lot of administrative work.	"The administration"<br>"Complicated issues with the tax authorities"<br>"I have a lot of admin work piling up that I can't get to"
Cognitive demands	The employee experiences cognitive tasks requiring concentration and problem-solving.	"A lot of information was provided and I had to really focus and hope I could keep up"<br>"I couldn't combine certain things anymore. It felt like parts of my brain weren't working together anymore"
Collaboration problems	The employee experiences conflicts in collaboration with colleagues, clients, or partners.	"A colleague was late for the preparation of a workshop we were giving together"<br>"Project progress stalls because people don't read what they've already committed to"<br>"People not keeping appointments as quickly as I need"
Complex tasks	The employee experiences complex work tasks.	"I have to handle difficult cases"<br>"Complex problems"<br>"Very difficult task"
Emotional demands	The employee feels that work affects their emotions.	"Two customer complaints"<br>"I had a mediation meeting and it was quite intense"<br>"First one-on-one with my new manager. Tension about how it would go"
Health issues	The employee experiences health problems that hinder their work.	"I think I was so tired today that I couldn't concentrate"<br>"I experienced stress because I'm menstruating"<br>"Persistent headaches"
Insufficient communication	The employee experiences ineffective communication or lacks necessary information.	"Couldn't communicate with a foreign colleague"<br>"Suddenly a full inbox with meetings and hassle"<br>"I was in a meeting where I felt the real questions weren't being addressed"
Insufficient leadership	The employee experiences little or no support from colleagues or supervisors.	"Limited managerial skills"<br>"A supervisor who can't handle criticism and doesn't look for team solutions"<br>"Supervisors not keeping promises"

Interpersonal conflict	The employee experiences work-related or personal conflicts with others at work.	"Colleagues being unfair"<br>"I had to work with a colleague for four hours this morning who gave me the finger two weeks ago"<br>"An unpleasant colleague"
Interruptions	The employee experiences work interruptions.	"Frequently interrupted by calls or people"<br>"Constant small questions and interruptions"<br>"People at the office stopping by unexpectedly"
Job insecurity	The employee feels uncertain about job security.	"I have three months to prove myself or I'll be reassigned"<br>"Uncertainty about the future"<br>"Concerns about the impact of announced budget cuts on contracts"
Lack of autonomy	The employee experiences little or no autonomy or decision-making power.	"Stress due to lack of autonomy over an upcoming accreditation I can't influence"<br>"A colleague trying to control my schedule"
Lack of clarity	The employee experiences unclear tasks or responsibilities.	"Some uncertainty about others' expectations"<br>"Tasks I'm not sure are mine"<br>"No clear task yet"
Lack of meaning or motivation or repetitive tasks	The employee has repetitive tasks, lacks motivation, or feels their work is meaningless.	"Felt a bit useless"<br>"It was mandatory to attend but not very useful"
Lack of psychosocial safety	The employee experiences an unsafe work environment or undesirable behavior.	"Verbal aggression from a client"<br>"Feeling like there's no space"<br>"Discrimination related to my neurodiversity"
Lack of social support	The employee experiences lack of help and appreciation from colleagues.	"Not sure if I'm working with the right colleagues toward my goal"<br>"Very difficult task I had to handle alone"<br>"Had to present/pitch without proper support or practice"
Long or unusual working hours	The employee experiences long workdays, overtime, or irregular hours.	"Very long workday of nine and a half hours"<br>"Tired from waking up at 4:45"<br>"Spent more than eleven hours at the office today"
Organizational changes	The employee experiences workplace changes as unfavorable, harmful, or stressful.	"A lot of unrest due to reorganization"<br>"Constant changes mean we keep repeating the same story"<br>"Stressful team meeting discussing budget cuts"
Performance expectations	The employee experiences high expectations from the organization, team, or colleagues.	"The targets are currently impossible"<br>"Had to present/pitch without proper support or practice"<br>"Feeling inadequate due to inexperience"
Personal stressors	The employee experiences stress or issues in their private life.	"I'm also a caregiver for my mother who lives with us"<br>"Pressure due to private situation"<br>"Stress from home that gave feelings of uncertainty and sadness that I had to park all day."
Planning problems	The employee experiences problems with work planning.	"Our schedules didn't align, which caused friction"<br>"Not enough time reserved"<br>"My schedule was overloaded today"

Problems with or lack of tools or resources	The employee lacks resources (tools, devices, instruments) to do their job well.	"Not enough materials, delivery delays from suppliers"<br>"IT problems with my laptop"<br>"Power outage caused delays"
Quantitative demands	The employee feels work cannot be completed on time or doesn't meet performance standards.	"Too much work"<br>"Too many meetings with clients, internally, and with applicants"<br>"Too many assignments"
Time pressure	The employee experiences high time pressure.	"Not enough time"<br>"Time pressure"<br>"Project with a rapidly approaching deadline"
Travel or commute burden	The employee finds travel time, business trips, or travel disruptions burdensome.	"Two-hour travel for a ten-minute presentation!"<br>"Lots of traffic both ways"<br>"Usually have to travel 1.5 to 2 hours one way"
Understaffing	The employee experiences a shortage of staff.	"Supervisor is sick, so all responsibilities fall on me"<br>"Still understaffed in our team"<br>"No replacement for a team member on maternity leave"
Work-Life imbalance	The employee struggles to balance work and private life due to conflicting demands.	"Feeling like I always have to be available"<br>"Taking care of parents and children in between"<br>"Work-life balance was off today"
Role-conflict	The employee experiences a role conflict due to conflicting expectations or demands.	"Many colleagues from different departments all think their task is the most important"<br>"Had to be creative in a situation but couldn't tell the truth, which goes against my principles"<br>"Stress from wanting to be human but also having to represent financial interests"
No stressors	It is explicitly stated that there were no issues.	"I didn't experience any issues at work today"<br>"None"<br>"Actually no issues"
No answer	No answer given.	""

Energy givers:

Table S2 Category system for coding open answers – energy givers

**Category**	**Description**	**Examples**
Accomplishment/ achievement	The employee experiences success in an inconcrete situation.	"I completed a few things and it gave me a sense of accomplishment"<br>"Checked off some items on my to-do list"<br>"Achieved a negotiation goal"
Appreciation and rewards	The employee experiences appreciation and recognition for efforts, achievements, and contributions at work.	"Appreciation from the manager"<br>"Had a performance review and received compliments"<br>"I appreciated that my manager valued my effort and work today and thanked me for it"
Autonomy	The employee experiences autonomy and decision-making power in their role.	"Control over my day"<br>"Deciding what to do myself"<br>"Able to organize my own work time and make decisions"
Catering/food/beverages	The employee mentions good food, drinks, or catering during a workday.	"Delicious cookies"<br>"A drink at the end of the day"<br>"Made a very tasty lunch"
Clarity and communication	The employee experiences effective and clear information exchange within the organization.	"Smooth communication"<br>"Things were well explained"<br>"Had a good conversation with the supplier and clarified where the problems are"
Helping others	The employee supports, helps, or coaches others at work.	"Guiding a colleague in her development gave me energy today"<br>"Helping colleagues so they can move forward"<br>"I got energy from coaching a junior colleague today"
Collaboration	The employee experiences effective collaboration within the team or with clients and partners.	"Able to collaborate, brainstorm, and create with a colleague for a few hours"<br>"Good discussions at our retreat"<br>"Good conversation with developers about further development"
Sport and exercise	The employee mentions sports or physical activity.	"I went for a run in between"<br>"I cycled during the break"<br>"Got energy from a nice walk outside"
Concentration/ focus	The employee experiences concentration or a flow state during work.	"I was just in a good flow today"<br>"No meetings today so I could work calmly and focused on two assignments"<br>"I was able to work focused"
Creativity/ inspiration/ innovation	The employee finds the work creative, innovative, or inspiring.	"Brainstorming about new possibilities"<br>"Thinking and being creative"<br>"Had a substantial idea to make knowledge available in practice"
Feedback	The employee receives feedback on work performance and areas for improvement.	"My project leader mentioned that what I delivered last week was of great value to the project"<br>"A very positive evaluation with a client"<br>"The performance review gave me insights I didn't have before about my results"
Flexible work time and location	The employee experiences flexibility in workplace or working hours.	"I worked from home today, so I could also do some laundry, which saves me time on the weekend"

		"Worked from home, so I could start calmly in my sweatpants and do some laundry in between"<br>"Nice that I can work hybrid and flexibly, so I can plan private appointments in between"
Humor/ enjoyment	The employee finds humor and jokes positive.	"A few jokes and gallows humor to handle the emotional material"<br>"Conversations with participants and unexpected jokes"<br>"Good atmosphere and humor in a work meeting"
Job content/ work tasks	The employee finds the work content interesting.	"The content of my work"<br>"Content-wise interesting work"<br>"I really enjoy the content of my work"
Leadership	The employee mentions an inspiring and supportive supervisor.	"A supportive supervisor"<br>"Mini team-building activity during work"<br>"Had a very good conversation with my manager about my tasks, and it was a very pleasant conversation"
Learning and development	The employee experiences opportunities to learn new things and develop themselves.	"Experience in knowledge sharing/intervision with my colleagues"<br>"Signed up for a new course"<br>"I learned about a topic I hadn't encountered before"
Meaningfulness	The employee finds the work or tasks meaningful.	"Meaningful work"<br>"Provided good therapies that people really benefited from"<br>"Did work that I find fulfilling"
Family and friends	The employee experiences positive interactions with family or friends.	"Lunch with my daughters"<br>"I talked to a friend on the phone today"<br>"Proud of my child's theater performance"
Positive atmosphere	The employee experiences a pleasant team atmosphere and feels comfortable.	"The pleasant atmosphere in the workplace"<br>"Good atmosphere in the department"<br>"Nice work atmosphere"
Problem solving	The employee feels satisfaction from solving a problem independently or collaboratively.	"Because I was able to solve issues at work"<br>"Discussed with the colleague I had a clash with this morning what we were facing"<br>"What gave me energy was turning impossibilities, problems, and obstacles into opportunities and improvements in the process"
Recovery time and activities	The employee mentions activities that help with relaxation or recovery.	"Short breaks to stretch my legs"<br>"Relaxed in the evening with a movie and sitting on the couch"<br>"I had some time to relax"
Social interrelations and activities	The employee experiences positive interactions and social activities with colleagues, clients, or supervisors.	"Nice customers at the checkout and friendly people"<br>"Talked to many team members about their autumn holidays, nice conversations"<br>"We had a team outing today"
Social support	The employee receives active support from colleagues or supervisors in tasks or difficult situations.	"Received support from colleagues"<br>"I felt I could be there for my employees"<br>"It was nice to work with my colleagues again and be supported by them"

Use of knowledge	The employee uses their professional knowledge and expertise in their work.	"I could use my knowledge and experience and felt useful"<br>"Shared knowledge"<br>"My expertise was called upon"
Vacation	The employee mentions planning or looking forward to a vacation.	"Last workday before vacation"<br>"I booked a week off, which was very nice"<br>"The day it was vacation was pleasant"
Variety	The employee experiences variety in tasks.	"Varied tasks"<br>"Variety and doing ad hoc things"<br>"Variety in my tasks"
Work environment	The employee finds the work environment conducive to pleasant, calm, or productive work.	"Working outside"<br>"Quiet at the office"<br>"Tidy workspace"
work life balance	The employee experiences the ability to combine work and private life during the day.	"Work-life balance. Despite having a sick child at home, I could manage because of the ability to work from home"<br>"At the end of my workday, I didn't have to work overtime"<br>"Hybrid working ensures an optimal work-life balance, which helped me a lot"
Self care	The employee practices self-care by paying attention to themselves and setting boundaries.	"Planned a relaxation moment with the masseur"<br>"Stopped working on time"<br>"Stopping work on time"
Low demands/ enough time	The employee has enough time to complete tasks and a calm workday.	"I could take it easy"<br>"It was just a calm workday"<br>"I had time to do my administration"
hobbies	The employee mentions their hobby as a way to relax or take a break from work.	"I sat in the sun with my book for 25 minutes this afternoon"<br>"Did some crafting, my hobby for some distraction"<br>"Painting after work"
Geen energiebronnen	It is explicitly stated that there were no energy sources.	"Actually not much"<br>"I didn't get energy from anything"<br>"None"
No answer	No answer given.	""
