# Supplementary material for: Using Semiautomated WhatsApp Messages for Daily Stress Measurements: Integrated Usability and Feasibility Study
Source: JMIR Form Res. 2026 Mar 11;10:e84032. doi: 10.2196/84032 (PMC12978546; doi:10.2196/84032)
Supplement: Multimedia Appendix 4 [file formative-v10-e84032-s004.pdf]

Multimedia Appendix 4 – Additional Tables

Table S1 Sample description based on pre-questionnaire (N=210) split by groups

		**group 1 (N=58)** *week 1: voice; week 2: text; emojis*		**group 2 (N=58)** *week 1: voice; week 2: text; no emojis*		**group 3 (N=57)** *week 1: text; week 2: voice; emojis*		**group 4 (N=37)** *week 1: text; week 2: voice; no emojis*	
		**n**	**%**	**n**	**%**	**n**	**%**	**n**	**%**
**Gender**	Female	51	87.9	45	77.6	43	75.4	31	83.8
	Male	7	12.1	11	19	14	24.6	6	16.2
	Other	0	0	2	3.4	0	0	0	0
**Age**	18 to 24 years	1	1.7	1	1.7	1	1.8	1	2.7
	25 to 34 years	8	13.8	14	24.1	11	19.3	11	29.7
	35 to 44 years	12	20.7	16	27.6	12	21.1	7	18.9
	45 to 54 years	25	43.1	18	31	14	24.6	12	32.4
	55 or older	12	20.7	9	15.5	19	33.3	6	16.2
**Education**	Low	2	3.4	0	0	2	3.5	0	0
	Medium	7	12.1	14	24.1	8	14	4	10.8
	High	49	84.5	44	75.9	47	82.5	33	89.2
**Workdays per week**	3 days	6	10.5	4	7	6	10.5	1	2.7
	4 days	23	40.4	25	43.9	25	43.9	19	51.4
	5 days	28	49.1	28	49.1	26	45.6	17	45.9
**Use of voice messages in everyday life**	Never	11	19	13	22.4	8	14	2	5.6
	Once in a while	24	41.4	15	25.9	20	35.1	14	38.9
	Sometimes	17	29.3	17	29.3	23	40.4	13	36.1
	Often	6	10.3	13	22.4	6	10.5	7	19.4

Table S2 Comparison of adherence depending on modality and use of emojis (adherence capped at 100%)

**Adherence (%)**	**Group 2 + 4 (N=94)** ***week 1: text; week 2: voice***		**Group 1 +3 (N=114)** ***week 1: voice; week 2: text***		**Wilcoxon rank test**	
	M	SD	M	SD	W	p
Week 1 (%)	94,26	11,07	85,57	23,5	6122	0,028
Week 2 (%)	84,52	25,36	74,91	35,24	5926	0,140
	**Emojis (N=114)**		**No emojis (N=94)**		**Wilcoxon rank test**	
	M	SD	M	SD	W	p
Total (10 days)	84,65	24,74	85,11	20,8	5144	0,592
